# Correlation between Quadriceps Endurance and Adduction Moment in Medial Knee Osteoarthritis

**DOI:** 10.1371/journal.pone.0141972

**Published:** 2015-11-05

**Authors:** Soon-Hyuck Lee, Jin-Hyuck Lee, Sung-Eun Ahn, Min-Ji Park, Dae-Hee Lee

**Affiliations:** 1 Department of Orthopaedic Surgery, Korea University College of Medicine, Anam Hospital, Seoul, Korea; 2 Department of Sports Medical Center, Korea University College of Medicine, Anam Hospital, Seoul, Korea; 3 Department of Orthopaedic Surgery, Samsung Medical Center, Sungkyunkwan University School of Medicine, Seoul, Korea; VIT University, INDIA

## Abstract

It is not clear whether the strength or endurance of thigh muscles (quadriceps and hamstring) is positively or negatively correlated with the adduction moment of osteoarthritic knees. This study therefore assessed the relationships between the strength and endurance of the quadriceps and hamstring muscles and adduction moment in osteoarthritic knees and evaluated predictors of the adduction moment. The study cohort comprised 35 patients with unilateral medial osteoarthritis and varus deformity who were candidates for open wedge osteotomy. The maximal torque (60°/sec) and total work (180°/sec) of the quadriceps and hamstring muscles and knee adduction moment were evaluated using an isokinetic testing device and gait analysis system. The total work of the quadriceps (*r* = 0.429, *P* = 0.037) and hamstring (*r* = 0.426, *P* = 0.045) muscles at 180°/sec each correlated with knee adduction moment. Preoperative varus deformity was positively correlated with adduction moment (*r* = 0.421, *P* = 0.041). Multiple linear regression analysis showed that quadriceps endurance at 180°/sec was the only factor independently associated with adduction moment (*β* = 0.790, *P* = 0.032). The adduction moment of osteoarthritic knees correlated with the endurance, but not the strength, of the quadriceps muscle. However, knee adduction moment did not correlate with the strength or endurance of the hamstring muscle.

## Introduction

Biomechanical factors are believed to play a role in the progression of knee osteoarthritis.[1] The effects of biomechanical factors on the progression of knee osteoarthritis may best be evaluated by measuring the dynamic load on the knee joint during walking,[2, 3] because mechanical overload of the joint has detrimental effects on the progression of knee osteoarthritis.[4, 5] The knee adduction moment is considered a surrogate measure of dynamic loading on the knee joint and is thought to increase as osteoarthritis becomes more severe.[6, 7]

The knee adduction moment has been shown to be higher in patients with knee osteoarthritis than in healthy individuals and to be related to the progression of medial knee osteoarthritis.[8, 9] Given these observations, and the fact that quadriceps strength and endurance are reduced in patients with osteoarthritic knees due to functional impairment and increased pain,[10, 11] it would seem that quadriceps strength and endurance should show a negative correlation with the knee adduction moment as the severity of knee osteoarthritis increases; that is, the greater the adduction moment, the weaker the quadriceps muscle. However, the adduction moment may show a positive correlation with quadriceps strength, because the quadriceps is the major abduction moment generator that stabilizes the loaded knee joint.[12, 13] Therefore, it remains unclear whether quadriceps strength and endurance are positively or negatively correlated with adduction moment in osteoarthritic knees. Additionally, few studies have analyzed the associations between the strength and endurance of the hamstring muscle in the knee joint and knee adduction moment.[12–14] The present study was therefore designed to evaluate the relationships between the strengths and endurances of the quadriceps and hamstring muscles and the adduction moment in osteoarthritic knees, as well as to identify predictors of the adduction moment during gait. It was hypothesized that quadriceps strength and endurance are negatively correlated with knee adduction moment in osteoarthritic knees.

## Materials and Methods

### Ethics Statement

The ethical approval of this study protocol was granted by Institutional Review Board of the Korea University Anam Hospital (permit no. AN11133-002). Written informed consent was obtained from all subjects before participation in this study.

### Study Design and Patient Enrollment

This cross-sectional study enrolled patients of both sexes with unilateral medial osteoarthritis and varus deformity who were candidates for open wedge osteotomy from May 2012 to October 2013. Muscle strength and endurance, as well as knee adduction moment during gait, were measured before open wedge high tibial osteotomy. Patients were excluded if they were ineligible for high tibial osteotomy or had symptomatic osteoarthritis of the patellofemoral joint and lateral tibiofemoral compartment, rheumatoid arthritis, knee range of motion <100°, grade ≥3 lateral collateral ligament laxity, or flexion contracture >10°. Patients with severe bilateral knee osteoarthritis (Kellgren-Lawrence grade ≥2 on the contralateral side) were also excluded.

All 39 patients (39 knees) initially approached agreed to participate in the study. On assessing eligibility, two patients were excluded, one with severe patellofemoral osteoarthritis and the other with 15° flexion contracture; hence, 37 patients (37 knees) were enrolled. Two other patients (two knees) could not undergo complete isokinetic strength tests due to severe knee pain. The final analysis therefore involved data from 35 patients (35 knees). The primary end-point of this study was to determine whether adduction moment was correlated with quadriceps muscle strength or endurance. In a pilot study of six subjects, the correlation coefficient between total work of extension at 180°/sec and peak knee adduction moment was found to be 0.512; hence, an priori power analysis was performed to assess the statistical significance of this correlation coefficient (0.512) between total work of extension at 180°/sec and peak external knee adduction moment, with alpha set at 0.05 and the power at 0.8. That analysis showed that 30 patients were required to demonstrate a statistically significant relationship between quadriceps endurance and adduction moment. The current study involved 35 patients, indicating adequate power (0.844) for detecting a significant correlation between the two variables.

### Isokinetic Test

Isokinetic knee strength was evaluated only in the arthritic limb using a Biodex multi-joint system 4 (Biodex Medical Systems, Shirley, NY). Subjects were seated on a dynamometer with their trunk perpendicular to the floor, and both the hip and knee flexed to 90°. The center of motion of the axis was aligned to the lateral femoral condyle.

The thigh of each subject was immobilized with a strap, and the dynamometer attachment was aligned to the lateral malleolus of the limb under test. The resistance pad was placed as distally as possible on the tibia while still allowing full dorsiflexion at the ankle.

The range of motion of the knee joint was set from full extension (0°) to flexion of 100°., Prior to testing, subjects performed a warm-up exercise consisting of 5 submaximal knee flexions and extensions of each leg at 60°/sec and 180°/sec, respectively. In the test session, subjects performed 5 isokinetic knee extensions and flexions of each leg at an angular velocity of 60°/sec and 15 at 180°/sec, with 30 seconds of rest between tests.[15, 16] The mean value of peak torque and total work from the 2 sets were used for analysis. Muscle strength was determined from measurements of the peak torque, i.e., the highest point on the torque curve normalized to each participant’s body mass(N·m/kg) recorded during flexion and extension at 60°/sec. Muscular endurance was obtained by measuring the total work, defined as the total area under all torque curves in the test repetitions (J) for flexion and extension at 180°/sec. Strength and endurance measured during extension were attributed to the quadriceps, whereas those during flexion were attributed to the hamstring.

The balance between the maximal strengths of the hamstring and quadriceps muscles was determined by measuring the hamstring-to-quadriceps strength (HQ) ratio, which may reflect the agonist-antagonist strength relationship about the knee joint. [17, 18] Gravity corrections were applied according to the specifications of the equipment manufacturer, and data processing was performed using Biodex Advantage Software 3.0 (Biodex Medical Systems Inc, Shirley, NY, USA).[16, 19–21]

### Radiographic Measurements

Varus deformities were recorded by measuring the mechanical axis (MA) on full-length standing radiographs, using a picture archiving and communication system (PACS, PI View STAR, version 5025; Infinitt, Seoul, Korea). The MA was defined as the angle subtended by a line drawn from the center of the femoral head to the center of the knee and a line drawn from the center of the knee to the center of the talus. MA was independently evaluated by two orthopedic surgeons with significant experience in the field. Each surgeon measured the MAs of each of the 35 patients twice, with intervals of 2 weeks between measurements. The average of the measurements by the two surgeons at both times was used in the analyses.

### Evaluation of Knee Adduction Moment

Marker data were obtained using six high-speed cameras (60 frames/second, Hawk Digital Camera System, Motion Analysis Corp.).[22] Data on the ground reaction force (GRF) were calculated using two force platforms (sampling frequency 600 Hz: AMTI OR6-7, Advanced Mechanical Technology Inc.) placed at the center of the 8 m walkway, level with the ground. All patients performed walking trials in their own comfortable shoes at a self-selected walking speed and were instructed to place their foot accurately on the force platform when walking. Reflective markers were placed on the acromion process of the shoulder, the anterior superior iliac spine, the superior aspect of the L5-sacral interface, the lower thigh below the mid-point, the lateral femoral condyles, the lower shank below the mid-point, the lateral malleolus, the posterior calcaneus, and the center of the foot between the second and third metatarsal heads, with the latter at the same height from the floor as the heel marker. Inverse dynamics analyses were used to calculate knee adduction moment from the motion and force data.[23] Force was calculated from the position of the markers and the measurements of each limb segment angle and moment (hip, knee, and ankle), but except moment of upper body segment, due to augmentation of lower body segment moment may occur by the arm swing. The force data were filtered through a Butterworth low-pass digital filter with a cutoff frequency of 50 Hz. Peak external knee adduction moment was normalized by body weight multiplied by height, and data from six stride lengths on the injured side were collected during walking.

### Statistical analysis

The isokinetic muscle strengths and adduction moments during gait were tested twice in ten participants with a one week interval between tests. Radiographic measurements were also performed twice for the MA of all subjects, with an interval of 2 weeks between measurements. To quantify the test-retest reliability of isokinetic strength (peak torque and total work values of quadriceps and hamstrings), radiographic measurements (MA), and adduction moment, the intraclass correlation coefficient (ICC_3,1_) was used. The ICC_3,1_ and standard error (SE) of isokinetic peak torque were 0.83 and 1.35 for the quadriceps and 0.89 and 1.12 for the hamstring muscles. The ICC_3,1_ and SE of total work were 0.79 and 2.12 for the quadriceps and 0.82 and 1.68 for the hamstring muscles. In addition, the test-retest reliabilities were good for radiographic measurements of MA (ICC_3,1_ = 0.93, SE = 1.11) and adduction moment(ICC_3,1_ = 0.78, SE = 2.54).

Statistical analyses were performed using IBM SPSS Statistics software (version 20; IBM, Armonk, NY). Dataset used in this study is given in S1 Dataset. All parameters, including isokinetic test, MA, and adduction moment data, were shown to be normally distributed by the Kolmogorov-Smirnov test. The correlations of adduction moment with isokinetic data and preoperative varus deformity were assessed using Pearson correlation analysis. Multiple linear regression analysis was performed to identify predictors of knee adduction moment. The results were assessed for multicollinearity by examining the variance inflation factors (VIFs) of the predictor variables. If one variable had a VIF >10, multicollinearity was indicated,[24] and the variable was removed from the multiple linear regression analysis due to its possibility of being a confounding factor. *P* values < .05 were considered statistically significant.

## Results

### Radiographic results, isokinetic muscle strength, and adduction moment

Table 1 shows the demographic and clinical characteristics of the patients. The 35 patients included 12 males and 23 females, of mean age 57.8 years (range, 43 to 75 years). The mean MA was varus 8.8° ± 4.2° (range, 4° to 17°,).

**Table 1:** Demographic and clinical characteristics of subjects with knee osteoarthritis of the medial compartment.

	Medial OA
Gender (male/female)	12/23
Age (years)	57.8 ± 9.8 (45 to 70)
Height (cm)	162.3 ± 10.8 (148 to 186)
Body mass index (kg/m^2^)	25.2 ± 3.8 (18.6 to 33.3)
varus deformity (°)	8.8 ± 4.2(4 to 17)

Variables are described as mean ± standard deviation (range). OA, osteoarthritis

The mean peak torques of the quadriceps and hamstring muscles at 60°/sec were 64.2 ±27.8 N∙m/kg and 45.8 ± 25.3 N∙m/kg, respectively. Mean total work values of the quadriceps and hamstring muscles at 180°/sec were 621.0 ± 287.7 J and 412.4 ± 434.9 J, respectively. Thus, the average hamstring-to-quadriceps ratios were 67.2 ± 22.4% at 60°/sec and 61.9 ± 50.6 at 180°/sec, and the mean peak adduction moment was 1.25 ± 0.43.

### Correlations with and predictors of adduction moment

The isokinetic test parameters included six parameters related to strength and endurance of the thigh muscles and HQ ratios at 60°/sec and 180°/sec. Endurances (total work at 180°/sec) of the quadriceps (*r* = 0.429, *P* = 0.037) and hamstring (*r* = 0.426, *P* = 0.045) muscles were positively correlated with knee adduction moment. Adduction moment, however, did not correlate with the isokinetic strengths (peak torques at 60°/sec) of the quadriceps and hamstring muscles. The varus deformity was positively correlated with adduction moment (*r* = 0.421, *P* = 0.041, Table 2). Multiple linear regression analysis of factors associated with knee adduction moment included isokinetic parameters, such as the isokinetic strengths and endurances of the quadriceps and hamstring muscles, and varus deformity, either because these variables showed significant association with knee adduction moment on univariate analysis or were suggested to be possible predictors in previous studies.[7, 10, 25, 26] Multiple linear regression analysis showed that isokinetic endurance at 180°/sec of the quadriceps muscle was the only factor independently associated with knee adduction moment (*β* = 0.790, *P* = 0.032, Table 3).

**Table 2:** Correlation between adduction moment and demographic characteristics, muscle strength and endurance, and varus deformity of arthritic knees.

	Adduction moment (arthritis side)	
Parameters	Correlation coefficient	*p*-value
Peak torque of quadriceps at 60°/sec (N∙m/kg)	0.113	0.600
Peak torque of hamstring at 60°/sec (N∙m/kg)	0.331	0.114
hamstring-to-quadriceps ratio 60°/sec (%)	0.146	0.496
Total work of quadriceps at 180°/sec (J)	0.429	**0.037** *
Total work of hamstring 180°/sec (J)	0.426	**0.045** *
hamstring-to-quadriceps (HQ) ratio 180 °/sec (%)	0.348	0.096
Mechanical axis(°)	0.421	**0.041** *

*P< 0.05

**Table 3:** Multiple linear regression analysis of predictors of the adduction moment of the involved limb in patients with medial knee osteoarthritis.

Dependent variables	Independent Variables	Unstandardized coefficients		Standardized coefficients	
		B	SE (B)	β	P-value
Adduction Moment	Peak torque of quadriceps at 60°/sec (N∙m/kg)	-0.003	0.002	-0.492	0.130
	Peak torque of hamstring at 60°/sec (N∙m/kg)	-0.001	0.003	-0.242	0.622
	Total work of quadriceps at 180°/sec (J)	0.001	0.002	0.790	**0.032** *
	Total work of hamstring at 180°/sec (J)	-0.000	0.000	0.331	0.488
	Mechanical axis (°)	0.021	0.010	0.473	0.078

*P< 0.05

## Discussion

This study evaluated the relationships between the strength and endurance of the quadriceps and hamstring muscles and adduction moment in 35 medial osteoarthritic knees and assessed predictors of the adduction moment. The most important finding of this study was that total work of the quadriceps measured at 180°/sec was the only predictor of knee adduction moment in osteoarthritic knees.

The relationships of quadriceps strength and endurance with knee adduction moment in osteoarthritic knees remain poorly understood. Agreeing with our results, recent studies also found no correlation between knee extensor strength and knee adductor moment. [27, 28]A previous study assessed the relationship between quadriceps strength and knee adduction moment in patients with osteoarthritis. That study, which measured quadriceps strength and peak knee adduction moment during walking in 184 subjects with medial knee osteoarthritis, found no significant association between quadriceps strength and peak knee adduction moment.[28] Another more recent study determined the extent to which knee extensor strength and power were related to the knee adduction moment. That study also showed that knee adduction moment was not related to knee extensor strength, but was related to knee extensor power. [27]Although no correlation was observed between knee extensor strength and adduction moment in the present study, a close correlation was found between quadriceps endurance and knee adduction moment. Direct comparison of the results of the two earlier studies mentioned above with those of the present work is made difficult by the different measurement variables, such as isokinetic strength, power, and endurance, used in the studies. Several factors could have given rise to the different results among the studies, such as inconsistencies in the relationship between isokinetic muscle measurement variables and adduction moment. One of the earlier studies was a retrospective evaluation of 3 separate cohorts of 184 community volunteers with medial knee osteoarthritis. These cohorts were evaluated for three purposes, namely the effects on knee adduction moment of footwear, lateral insoles, and quadriceps strengthening. In contrast, our study recruited candidates scheduled for open wedge high tibial osteotomy, indicating that their clinical symptoms were more severe. In addition, although the two earlier studies measured isometric muscle strength, our study measured both isokinetic muscle strength at 60°/sec and endurance at 180°/sec. Isometric strength may not reflect the physiologic knee condition, because the load forces used during isometric strength tests are greater than those applied during in vivo physiologic loading of osteoarthritic knees during daily life activities, and also higher than those used in isokinetic strength tests.[28] Differences in gait pattern and pain severity may also have affected values obtained for adduction moment and quadriceps strength. The first study mentioned above[28] measured walking speed and knee pain to control for their potential influence on the knee adduction moment. However, that study did not evaluate the foot progression angle, which also affects the knee adduction moment. The second study mentioned above[27] and our study did not measure the walking speed, foot progression angle, and magnitude of knee pain. Therefore, direct comparison of the results of these studies is difficult.

Contrary to our hypothesis, quadriceps muscle endurance was positively correlated with knee adduction moment and was a positive predictor of knee adduction moment in osteoarthritic knees. These relationships mean that the greater the quadriceps endurance the greater the knee adduction moment. Adduction moment is affected by the magnitude of the GRF passing medial to the knee joint center during gait and the moment arm of the GRF, defined as the perpendicular distance from the GRF to the center of the knee joint.[14] Patients with greater quadriceps strength and/or endurance may walk faster,[29] and may, in turn, have greater magnitudes of the GRF and greater knee adduction moment.[9, 30] Furthermore, reduced pain resulting from increased quadriceps strength may also increase the knee adduction moment, [31] despite the inconsistent relationship between knee pain severity and adduction moment.[8, 32]

In addition, it is possible that the increased knee adduction moment of osteoarthritic knees may contribute to an increase in quadriceps strength or endurance. The quadriceps can generate considerable force due to its large cross-sectional area, thus exerting a relatively large knee abduction moment. An increase in quadriceps strength may be a compensatory mechanism to stabilize the knee joint by counteracting the increased knee adduction moment of osteoarthritic patients.[33, 34]However, long-term, prospective follow-up studies are needed to elucidate the causal relationship between quadriceps strength or endurance and knee adduction moment in osteoarthritic knees.

Interestingly, the results of the present study showed that knee adduction moment was not associated with peak torque of the quadriceps measured at 60°/sec, but was only related to and affected by the total work of the quadriceps measured at 180°/sec. The peak isokinetic muscle strength measured in this study may not correspond to actual muscle contraction or activation level while walking, because humans do not exhibit maximal contractions of their knee extensors and flexors muscles while walking. Rather, they use an individual-specific proportion of their maximal strength, generally below maximum limits. However, peak isokinetic knee muscle strength is a clinically reliable measure and less burdensome to participants.[35] Therefore, most previous studies measured only peak muscle torque at 60°/sec.[36–38] Our findings suggest that the total work of muscles at 180°/sec should be included in isokinetic tests of muscles around the knee joint, especially when evaluating the association between thigh muscles and knee adduction moment. Peak muscle torque at 60°/sec may not be the most valid measure when investigating the relationship between muscle strength and knee adduction moment during gait.

This study had several limitations. Other factors that could affect knee adduction moment, such as foot progression angle, knee flexion angle, and walking speed, were not evaluated. Out-toeing gait could decrease knee adduction moment,[39]whereas increased knee flexion angle and higher walking speed have been reported to increase knee adduction moment.[9, 30, 40] However, none of our patients had flexion contractures of the knee joint and their walking speeds (approximately 1 m/s) during gait analysis did not differ markedly from those in previous studies.[28, 41] This study only included patients with preoperative high tibial osteotomy, medial knee osteoarthritis and concomitant varus deformity. The varus deformities in our subjects may have been a confounder, affecting the close relationship between the quadriceps muscle and adduction moment in our study. A greater varus malalignment could result in a higher adduction moment and a stronger quadriceps muscle,[26, 33] although varus deformity was not found to be a predictor of knee adduction moment in our study. Nevertheless, this confounding effect of varus deformity could be ruled out, because multicollinearity was not observed during the regression analysis. Another limitation was that muscle strength and endurance were measured only for knee extensors (quadriceps) and flexors (hamstrings). Hip abductors and adductors may be associated with knee adduction moment by controlling the center of mass position and mediolateral acceleration.[42–44] The gastrocnemius may also help stabilize knee adduction moment.[13] Finally, we measured the parameters only on the side of the osteoarthritic knee, not on the contralateral side or in a control group. Knees on the contralateral side in patients with osteoarthritis tend to be osteoarthritic. Aberrant afferent information in the injured knee may affect muscle strength in the contralateral uninjured limb, with these individuals showing impairments in bilateral muscle strength.[45] This may reduce the reliability of contralateral knee measurements as a control group. However, future research should examine whether these correlations also occur in asymptomatic subjects and in the contralateral limb of patients with knee osteoarthritis.

## Conclusions

In conclusion, the knee adduction moment during gait in osteoarthritic knees was positively correlated with the endurance of the quadriceps muscle, but not with the strength. However, knee adduction moment was not correlated with the strength or endurance of the hamstring muscle.

When evaluating the relationship between isokinetic values of the thigh muscle and knee adduction moment in patients with osteoarthritic knee, the total work of muscles at 180°/sec (endurance) should be included in isokinetic tests. Further investigations are warranted to determine how the relationship between quadriceps endurance and knee adduction moment affects the progression of patients with knee osteoarthritis.

## Supporting Information

S1 Dataset Raw data used in this study. (XLSX)
